# EXPLORING MODELS OF CARE INTEGRATION TO ADDRESS THE NEEDS OF PERSONS AGING WITH HIV

**DOI:** 10.1093/geroni/igad104.0378

**Published:** 2023-12-21

**Authors:** Abigail Baim-Lance, Gemmae Fix, Thomas Laskow

## Abstract

By 2035, the majority of persons living with HIV will be 50 and older. The clinical and social needs of this population have been well documented, including the negative impacts of persistent stigma on quality of life and health outcomes. Responding to such needs requires integrated, patient-centered approaches to care and supportive services. Care integration is defined as the bringing together of two or more previously unlinked services. Care integration is used within and outside of the HIV care settings, but how such models should be designed or adapted for older persons with HIV (OPWH) is less certain given their unique needs. This symposium will explore case studies representing different models of care integration developed within and outside of HIV care settings, their relevance to OPWH, evidence of implementation including known barriers and facilitators, and opportunities to advance such models. Our first presentation describes an embedded geriatrics outpatient consultation service within an HIV care setting in New York City (Condo). Our second presentation focuses on the barriers and facilitators to the integration of substance use treatment into HIV care in Georgia (Bender). Our third and fourth presentations will describe service models developed outside of HIV care, which are now being applied to support OPWH including the Veterans Affairs patient-centered Whole Health approach (Fix) and the CAPABLE program to support aging in place (Smith). With our discussant, we will reflect on these models of integration to enhance care and support systems for this population.

